# Quercetin Alleviates Toxicity Induced by High Levels of Copper in Porcine Follicular Granulosa Cells by Scavenging Reactive Oxygen Species and Improving Mitochondrial Function

**DOI:** 10.3390/ani13172745

**Published:** 2023-08-29

**Authors:** Nannan Qi, Wenwen Xing, Mengxuan Li, Jiying Liu

**Affiliations:** School of Biotechnology, Jiangsu University of Science and Technology, Zhenjiang 212018, China; qinannan416@163.com (N.Q.); xingwenwen0302@163.com (W.X.); 212211801208@stu.just.edu.cn (M.L.)

**Keywords:** porcine, copper toxicity, granulosa cells, oxidative stress, reproductive performance, mitochondria

## Abstract

**Simple Summary:**

CuSO_4_ is a commonly used growth-promoting agent in pig production. It is clear that long-term intake of excess CuSO_4_ will damage the reproductive performance of pigs. To better understand the toxic mechanism of CuSO_4_ and the alleviating effect of quercetin on it, the oxidative stress level and mitochondrial status of porcine follicular granulosa cells treated with CuSO_4_, or quercetin combined with CuSO_4_ were investigated in this study. Our results suggest that quercetin could alleviate toxicity induced by high levels of copper in porcine ovarian granulosa cells by scavenging ROS and improving mitochondrial function.

**Abstract:**

CuSO_4_ is the most commonly used feed additive in pig production at present, but long-term ingestion of excessive copper would lead to chronic copper toxicity. High copper could reduce the reproductive efficiency of sows and seriously affect the development of the pig industry. Quercetin (QUE), a powerful antioxidant, reduces toxicity of a number of heavy metals. Porcine granulosa cells (pGCs) are crucial to the fate of follicle development. The present study found that high concentrations of CuSO_4_ induced ROS production, which resulted in decreased mRNA expression of antioxidant-related genes *GPX4*, *CAT*, and *SOD2* and increased mRNA expression of *SOD1*, *TRX*, and *HO-1*. The protein expression of antioxidant enzymes SOD2 and HO-1 decreased. Moreover, the concentration of MDA increased, the activity of CAT decreased, and the content of GSH decreased. After high copper treatment, the mitochondrial membrane potential (MMP) was decreased and the morphological structure was changed. However, the combined treatment with Quercetin (QUE) reversed these changes, and the level of cellular oxidative stress decreased. Therefore, we conclude that high copper has oxidative toxicity to pGCs, and QUE could remove the ROS induced by high copper, protect mitochondria from oxidative stress damage, and improve the function of pGCs.

## 1. Introduction

Copper is an essential trace element in the body and plays an important role in the growth and development of human and animals [1]. It could be absorbed by the gastrointestinal tract, lungs, and even the skin, entering the systemic circulation, and accumulating in organs and tissues. CuSO_4_ is also an important trace element additive required for animal feed. It is widely used as a growth promoter and insect repellent in actual production [2,3]. However, excess copper will lead to cytotoxicity [4], and the recent literature reports cuproptosis mainly due to proteotoxic stress caused by aggregation of lipated proteins and loss of iron–sulfur cluster proteins [5]. At the same time, a large number of studies have shown that one of mechanisms of copper toxicity is oxidative stress [6,7,8,9,10,11]. When high levels of copper act on the ovaries, it will affect the reproductive performance of animals [12,13]. Part of copper enters the organism as structural cofactor of superoxide dismutase (SOD), ceruloplasmin, and metallothionein to regulate the redox balance [14], and the other part of free copper ions has oxidative activity and could catalyze the production of reactive oxygen species (ROS) through the Fenton reaction [13,15]. Excessive ROS leads to changes in mitochondrial morphology, a decrease in MMP [9], and eventually induces mitochondrial dysfunction and apoptosis [7,16,17], which further accelerates the damage induced by high copper. However, the mechanism of copper toxicity on the reproductive performance of pigs is still unclear. In addition, screening for a natural ingredient that alleviates oxidative stress caused by high copper may be an effective protective strategy.

QUE is a flavonol compound with a variety of biological activities, which could not only chelate divalent copper ions [18], but also scavenge free radicals. It is mainly found in vegetables and fruits such as broccoli, apples, onions, etc. As an exogenous antioxidant, it may function with the endogenous antioxidant defense system [19]. Moreover, it could promote zinc into cells as an intracellular antioxidant and enhance antioxidant capacity [18]. Studies have shown that QUE could alleviate the toxic effects of the heavy metal cadmium on the kidney [20,21] and testis [22] of rats. In addition, QUE could improve the antioxidant capacity of aged mice, improve the quality of oocytes [23], improve the antioxidant capacity of menopausal rat ovaries [24], and alleviate oxidative stress in porcine [25] and rat [24] ovarian granulosa cells. Moreover, QUE alleviates the toxicity of excess copper has been described in mouse hepatocytes [26], but not in pGCs. Therefore, we hypothesized that oxidative stress induced by high copper was involved in the reproductive toxicity of high copper to sows, which could be alleviated by QUE.

In this study, pGCs were used as target cells to investigate the toxic mechanism of high copper. The oxidative damage model of pGCs induced by high copper was established by qPCR, WB, oxidative index detection, and ROS staining. JC-1 staining, mitochondrial morphological staining, MitoSOX staining, and transmission electron microscopy were used to detect the damage of mitochondria and the content of mitochondrial reactive oxygen species (mtROS) induced by high copper. Similarly, the mechanism of QUE protects pGCs from oxidative stress induced by high copper was determined by examining the antioxidant levels and mitochondrial oxidative stress and so on. This research has an important practical implication for pig production. It is beneficial to alleviate the common copper toxicity in production, improve the antioxidant properties of pGCs, and protect their normal function, which may reduce follicular atresia and increase the farrowing rate.

## 2. Materials and Methods

### 2.1. Isolation and Culture of pGCs

The animal experimental protocols were submitted to the Institutional Animal Care and Use Committee (IACUC) of Jiangsu University of Science and Technology (G2022SJ12, 2022.03, Zhenjiang, China) for review and approval. Animal care and handling were in accordance with IACUC guidelines.

The fresh ovaries were from healthy 6-month-old sows slaughtered in the slaughterhouse. The sows weighed approximately 110–120 kg, and all feeding conditions were normal before slaughter. After the pigs were killed by electric shock, about 70 ovaries were collected and stored in normal saline containing 1% penicillin and streptomycin (Beyotime Biotechnology, Shanghai, China) at 37 °C, and sent to the laboratory within 2 h. After five alternating washes with 75% alcohol and normal saline containing 1% penicillin and streptomycin, the follicles were immersed in normal saline. Follicular fluid from (3~5 mm diameter) healthy follicles was taken using 22-gauge needles and centrifuged at 800× *g* for 5 min. The supernatant was discarded and the cells were washed twice with DMEM/F12 (Life Technologies Co., Carlsbad, CA, USA) and centrifuged at 800× *g* for 5 min. In the end, the cells were seeded in DMEM/F12 containing 10% fetal bovine serum (Gibco, Courtaboeuf, France) and 1% penicillin-streptomycin in culture bottles and placed in an incubator containing 5% CO_2_ at 37 °C for 48 h. Then the cells were washed three times with phosphate-buffered saline (PBS), added to DMEM/F12 containing 10% fetal bovine serum and 1% penicillin-streptomycin, placed in an incubator containing 5% CO_2_ at 37 °C for 24 h, and seeded into the desired well plates. All the cells used in the experiments were primary culture cells.

CuSO_4_·5H_2_O of ≥99% purity (CAS No. 7758-99-8, Duly, Nanjing, China) was added to double-distilled water to form 1 M storage solution and used according to 1:1000 dilution with complete medium into 1 mM diluent, which was then configured to the final concentration required for the experiment using complete medium. In the experiment of copper toxicity model induced by QUE, QUE (CAS No. 117-39-S, Yuanye, Shanghai, China) with purity ≥97% was dissolved in dimethyl sulfoxide (Sangon Biotech, Shanghai, China) and prepared into 100 mM storage solution. When used, the complete medium was diluted into 100 µM diluent at a ratio of 1:1000. In the end, the diluent was added into the complete medium to prepare the working concentration required for the experiment.

### 2.2. Cell Viability Assay

To assess the toxicity of different concentrations of CuSO_4_ on pGCs, the cell viability of pGCs treated with different concentrations of CuSO_4_ was measured by CCK8 assay (Sangon Biotech, Shanghai, China). Cells were counted and seeded into 96-well plates at a density of 5000 cells/well and grown for 24 h. After treatment with drugs for the indicated time, the original medium was removed and 110 µL DMEM/F12 containing 10 µL CCK8 was added. After incubation in an incubator containing 5% CO_2_ at 37 °C for 2 h, the absorbance value at 450 nm was measured using a microplate reader. All reactions were repeated six times (*n* = 6).

### 2.3. Real-Time Fluorescence Quantification

To examine the mRNA expression of antioxidant genes in pGCs, total RNA was extracted from drug-treated cells using RNAiso Plus (Taraka, Dalian, China) according to the manufacturer’s instructions. And cDNA was synthesized using a reverse transcriptase kit (Taraka, Dalian, China). A total of 1 μL cDNA was used for qPCR system. For qPCR, the primers were selected, and slope of ∆CT vs. log input was <0.1. The efficiency of qPCR was 90% to 110%, and the Cq value of the negative control was greater than 38. The efficiency of qPCR and the Cq value of the negative control were within reasonable limits. The reaction system was added according to the instructions for use of the Cham Q SYBR qPCR Master Mix (Vazyme, Nanjing, China). The qRT-PCR reaction procedure is listed in Table 1, and the primers are listed in Table 2. Melting curve data were analyzed to determine PCR specificity. Relative expression of genes was analyzed by the 2^−ΔΔCt^ method using the Cq value of GAPDH as an internal reference for each sample. All reactions were repeated three times (*n* = 3).

### 2.4. Western Blot

RIPA lysate (strong) (Beyotime Biotechnology, Shanghai, China) with a ratio of 100:1 to protease inhibitors was prepared and precooled at 4 °C. At the end of treatment, the cells were washed three times with precooled PBS, and 100 µL of protein lysate was added to 12-well plates and incubated for 30 min on ice. At the end of incubation, the samples were collected. After centrifugation at 12,000× *g* for 15 min at 4 °C, the supernatant was removed for determination of total protein concentration using the BCA Protein assay kit (Beyotime Biotechnology, Shanghai, China). The concentration was adjusted with protease inhibitor lysates and then denatured with protein loading buffer (Beyotime Biotechnology, Shanghai, China). A total of 20 µg of protein was separated by on 4 to 20% SDS-PAGE preset gels (ACE Biotechnology, Nanjing, China) and transferred to PVDF membranes. After blocking the membranes with 5% skim milk for 1 h, the membrane was incubated at 4 °C with diluted primary antibodies SOD2 (1:1000, Proteintech Group, Wuhan, China), HO-1 (1:1000, Wanleobio, Shenyang, China), and TUBULIN (1:10,000, Bioworld Technology, St Louis Park, MN, USA) overnight. The next day, the primary antibody was removed and washed three times with 1 × TBST for 10 min, then the membrane was incubated with the secondary antibody for 1 h at room temperature and washed three times using 1 × TBST for 10 min. The negative control followed the same procedure but without the addition of the primary antibody. The enhanced ECL chemiluminescence detection kit (Yeasen, Shanghai, China) was used to observe the blots. The target proteins were visualized using a chemiluminescence imaging system (Qinxiang, Shanghai, China), and densitometry was performed using Image J 1.8.0 (National Institutes of Health, Bethesda, MD, USA). All bands are shown in the Supplementary Material. The relative protein expression was obtained by calculating the ratio of the band density of the target protein to the band density of TUBULIN. All reactions were repeated three times (*n* = 3).

### 2.5. Detection of ROS, MMP, mtROS, and Mitochondrial Morphology

Changes in intracellular ROS, MMP, mtROS, and mitochondrial morphology were assessed according to the manufacturer’s instructions. At the end of the treatment, the original culture medium was aspirated and treated with 2′,7′-dichlorofluorescein diacetate (DCFH-DA) (Beyotime Biotechnology, Shanghai, China), JC-1 working solution (Beyotime Biotechnology, Shanghai, China), MitoSOX (MedChemExpress, Shanghai, China) and Mito-Tracker Red CMXRos (Beyotime Biotechnology, Shanghai, China) were stained at 37 °C for 20 min. Then, cells were washed three times with DMEM/F12, and the ROS levels, MMP, mtROS, and mitochondrial morphology were observed by fluorescence microscopy (Olympus, Tokyo, Japan).

### 2.6. Determination of Oxidative Damage Parameters

At the end of treatment, the cells were scraped off using cell scrapers, and the supernatant was discarded after centrifugation at 1000× *g* for 10 min at room temperature. Next, 1 mL PBS was added to the cell precipitate for washing and the supernatant was centrifuged at 1000× *g* for 10 min to leave the cell precipitate. Then, the cell precipitate was washed twice. A total of 400 µL PBS was added to the cell precipitate and homogenized by blowing using a pipettor. The cells were crushed in an ice-water bath using an ultrasonic cell grinder (Scientz, Ningbo, China) with a power of 300 W, 3 s each sonication, 30 s intervals, and 5 repetitions. In the end, the supernatant was centrifuged at 12,000× *g* for 10 min at 4 °C, and the malonaldehyde (MDA), superoxide dismutase (SOD), glutathione (GSH), and catalase (CAT) were detected according to the instructions of commercial kits (Jiancheng, Nanjing, China). All reactions were repeated three times (*n* = 3).

### 2.7. Transmission Electron Microscopy Scanning

The ultrastructure of cells was observed using a JEM1400 transmission electron microscope (JEOL, Tokyo, Japan). Briefly, cells were harvested and immediately fixed in 2.5% glutaraldehyde at 4 °C, postfixed in 1% osmium tetroxide, dehydrated in graded ethanol, saturated and embedded in epoxy resin in a 1:1 ratio of propylene oxide, and sectioned for transmission electron microscopy.

### 2.8. Statistical Analysis

All experiments were repeated at least three times. GraphPad 8.0 statistical software (GraphPad Software, San Diego, CA, USA) was used for statistical analysis, and the results were expressed as mean ± standard error (SEM). Student’s *t*-test was used to compare the two sets of data. And one-way analysis of variance (ANOVA) followed by Tukey’s test was used to compare more than two groups. * *p* < 0.05 indicated significant difference, ** *p* < 0.01 indicated very significant difference, and ns indicated no significant difference.

## 3. Results

### 3.1. Changes in Cell Viability of pGCs Treated with Different Concentrations of CuSO_4_

**Figure 1:**
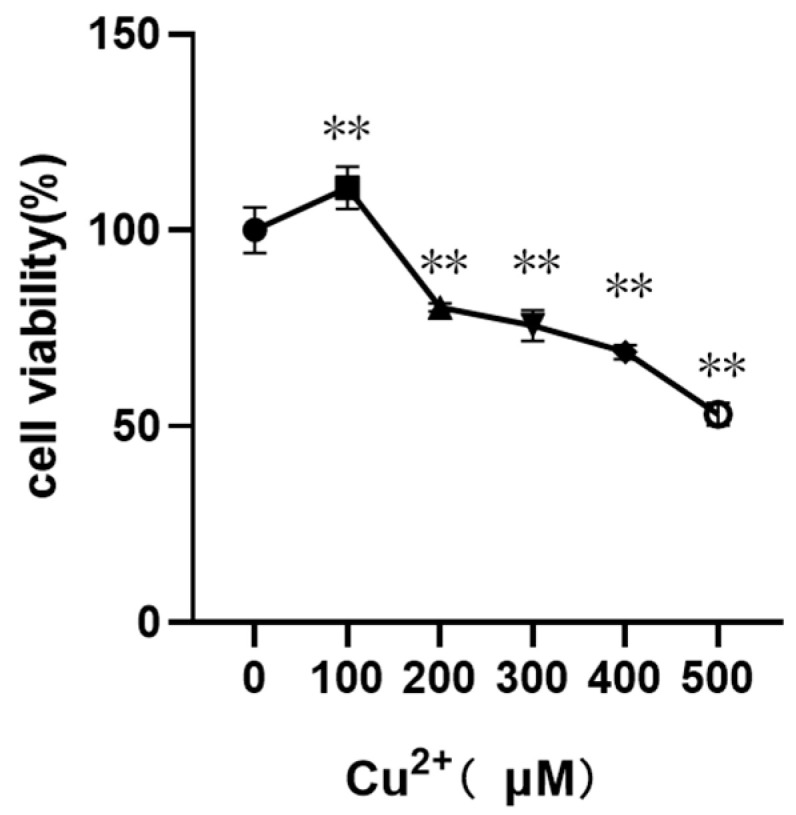
The cell activity changes of different concentrations of CuSO_4_ on pGCs. CCK8 kit was used to detect the viability of pGCs treated with CuSO_4_ at different doses (0, 100, 200, 300, 400, and 500 µM) for 24 h. ** *p* < 0.01, ** means extremely significant difference compared with the control group.

CCK8 assay was used to detect the cytotoxic effect of CuSO_4_ on pGCs treated with different concentrations of CuSO_4_ (0, 100, 200, 300, 400, 500 µM) for 24 h. The data were expressed as cell viability (%), and the results showed that with the increase in CuSO_4_ concentration, cell viability of pGCs decreased and the cytotoxicity increased. As shown in Figure 1, at CuSO_4_ concentration of 100 µM, the cell viability increased compared with the control group, indicating that low CuSO_4_ concentration was beneficial to the increase in cell viability. However, at CuSO_4_ concentration of 200 µM or more, the cell viability was extremely significantly decreased compared with the control group, indicating that high CuSO_4_ concentration was toxic to pGCs. In the following experiments, CuSO_4_ (100, 200 and 400 µM) was used to treat pGCs.

### 3.2. High Copper Induced Oxidative Stress in pGCs

**Figure 2:**
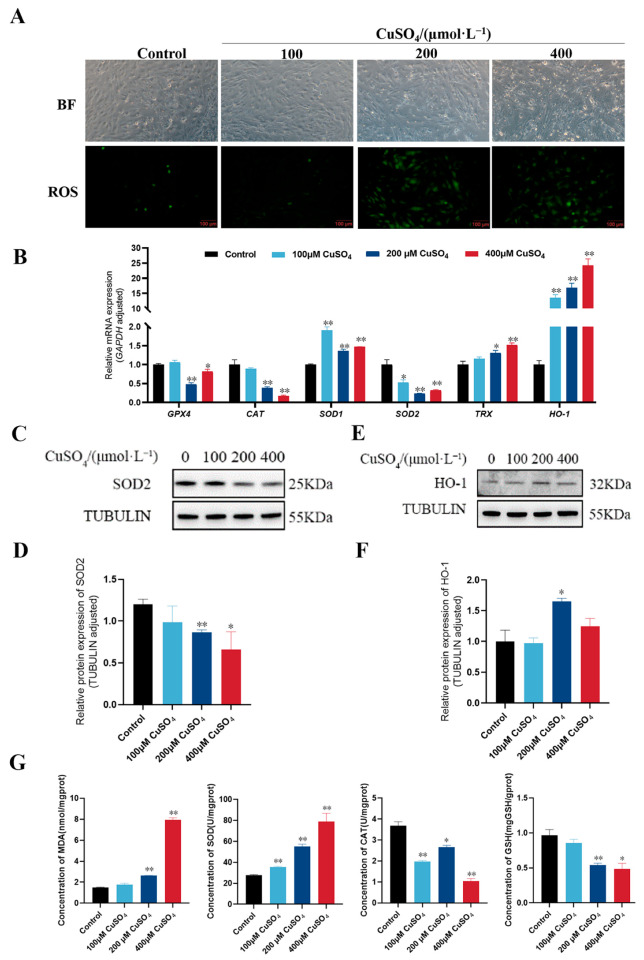
Effect of different concentrations of CuSO_4_ on oxidative stress in pGCs. (**A**) DCFH-DA fluorescence probe was used to detect the ROS level in pGCs after CuSO_4_ treatment for 24 h. Scale bar: 100 μm. (**B**) mRNA expression levels of antioxidant-related genes in pGCs treated with different concentrations of CuSO_4_ for 24 h. (**C**–**F**) The protein expression levels of antioxidant enzymes in pGCs treated with different concentrations of CuSO_4_ for 24 h. (**G**) The contents of MDA, SOD, CAT, and GSH in pGCs treated with different concentrations of CuSO_4_ for 24 h. * *p* < 0.05, ** *p* < 0.01, respectively, compared with the control group.

To explore the possible mechanism of CuSO_4_-induced cytotoxicity, the DCFH-DA probe was used to detect intracellular ROS levels after treatment with different concentrations of CuSO_4_ (0, 100, 200, and 400 µM). As shown in Figure 2A, the levels of ROS in cells treated with high concentrations of CuSO_4_ (200 and 400 µM) were significantly higher than control group, while the low concentration of CuSO_4_ (100 µM) did not cause change in ROS. Since high copper induces the generation of intracellular ROS, and the accumulation of ROS leads to oxidative stress, which reflects the level of intracellular oxidative stress. To further detect the level of oxidative stress, the mRNA (Figure 2B) and protein (Figure 2C–F) expression levels of antioxidant-related genes in the cells were detected. The results showed that CuSO_4_ at high concentrations (200 and 400 µM) significantly downregulated the mRNA expression of *GPX4*, *CAT*, and *SOD2*, and upregulated the mRNA expression of *SOD1*, *TRX*, and *HO-1*. Moreover, the protein expression of HO-1 was increased, and the protein expression of SOD2 was decreased. Overall, high concentrations of CuSO_4_ disturbed the redox balance within pGCs. After that, the intracellular levels of MDA, SOD, CAT, and GSH continued to be evaluated, and it can be seen from the results in Figure 2G that CuSO_4_ treatment increased MDA and SOD content and decreased CAT and GSH content. The results show that high copper increased the lipid peroxidation level and decreased the antioxidant level of pGCs, and in the end induced oxidative stress in pGCs.

### 3.3. High Copper Caused Mitochondrial Damage in pGCs

**Figure 3:**
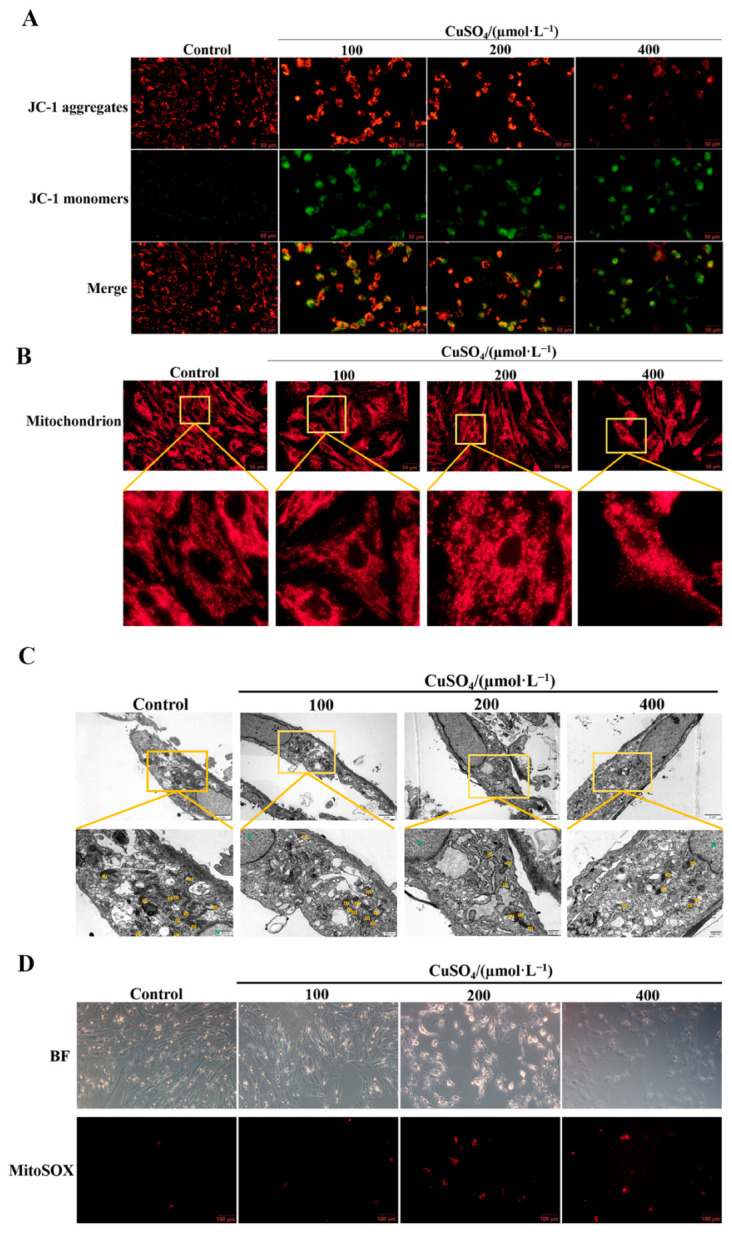
Effects of CuSO_4_ at different concentrations on mitochondrial oxidative stress and mitochondrial morphology in pGCs. (**A**) JC-1 fluorescent probe was used to detect the effect of CuSO_4_ on the MMP of pGCs. JC-1 polymer: red; JC-1 monomer: green. Scale bar: 50 µm. (**B**) Mitochondrial morphology of pGCs. Mitochondria were labeled with MitoTrackerTM. Scale bar: 50 µm. (**C**) CuSO_4_-induced mitochondrial ultrastructure changes in pGCs were detected by transmission electron microscopy. m represents mitochondria and N represents the nucleus. Scale bar: 2 µm; scale bar after magnification: 500 nm. (**D**) The level of mtROS after CuSO_4_ treatment was detected by MitoSOX fluorescent probe assay. Scale bar: 100 µm.

The pGCs treated with CuSO_4_ at different concentrations (0, 100, 200, 400 µM) for 24 h were labeled with fluorescent probe JC-1, and the changes in MMP were evaluated by fluorescence microscopy. Figure 3A shows that control cells showed higher levels of red fluorescence, while copper-treated cells showed higher levels of green fluorescence, indicating that the MMP was reduced after CuSO_4_ treatment. Next, the mitochondrial morphology was analyzed. The mitochondria in normal cells showed a reticular structure, which was significantly altered by high copper treatment compared with the control group. The mitochondria were destroyed and fragmented after treatment with high concentrations of CuSO_4_ (200, 400 µM) (Figure 3B). Meantime, analysis of the transmission electron microscopy results showed that CuSO_4_ exposure caused significant changes in mitochondrial morphology, manifested as mitochondrial swelling and rupture, disappearance of cristae, and obvious cell vacuolization (Figure 3C). In addition, ROS levels in mitochondria were increased after CuSO_4_ treatment (Figure 3D). These results suggest that high copper could induce mitochondrial oxidative damage in porcine pGCs.

### 3.4. QUE Alleviated pGC Toxicity Caused by Oxidative Stress Induced by High Copper

**Figure 4:**
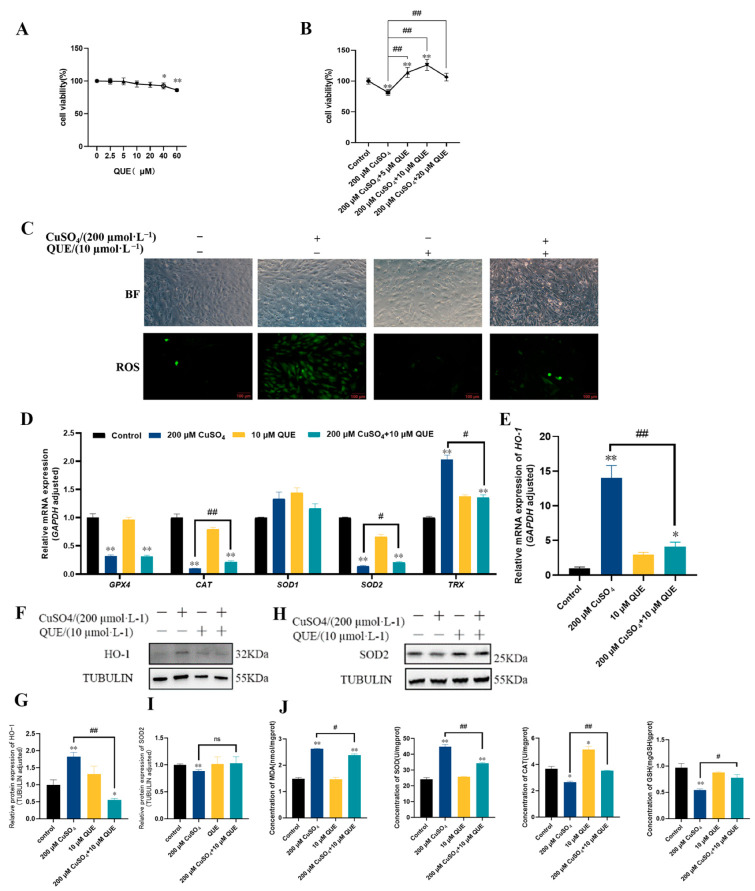
Effect of QUE supplementation on high copper-induced oxidative stress in pGCs. (**A**) CCK8 assay was used to detect the viability of pGCs treated with 0, 2.5, 5, 10, 20, 40, and 60 µM QUE for 24 h. (**B**) CCK8 assay was used to detect the cell viability of pGCs treated with 0, 5, 10, 20 µM QUE in combination with 200 µM CuSO_4_ for 24 h. (**C**) ROS levels in pGCs after combined treatment with 10 µM QUE and 200 µM CuSO_4_ for 24 h. Scale bar: 100 µm. (**D**,**E**) mRNA expression levels of antioxidant-related genes in pGCs after 24 h treatment with 10 µM QUE in combination with 200 µM CuSO_4_. (**F**–**I**) Protein expression levels of antioxidant enzymes in pGCs after combined treatment with 10 µM QUE and 200 µM CuSO_4_ for 24 h. (**J**) MDA, SOD, GSH, and CAT contents in the cells after combined treatment with 10 µM QUE and 200 µM CuSO_4_ for 24 h. * *p* < 0.05, ** *p* < 0.01, respectively compared with the control group; # *p* < 0.05, ## *p* < 0.01, respectively, compared with the 200 µM CuSO_4_-treated group; ns indicated no significant difference.

QUE, a natural and commonly used antioxidant, plays a significant role in a number of antioxidant experiments [27,28]. A literature search and our preliminary experiments (Figure 4A,B) indicated that cell viability was significantly decreased when the concentration of QUE was greater than 40 µM. Moreover, cell viability of the group treated with 200 µM CuSO_4_ and 10 µM QUE was the highest. Therefore, QUE at 10 µM was chosen as the treatment concentration for following experiments. After determining the concentration of QUE, the ROS changes in pGCs were examined, and the results shown in Figure 4C showed that the ROS levels in pGCs were significantly reduced after 24 h when combined treatment with 10 µM QUE and 200 µM CuSO_4_. Then, the mRNA (Figure 4D,E) and protein (Figure 4F–I) expression levels of antioxidant-related genes were detected, as well as the MDA, SOD, GSH, and CAT contents (Figure 4J). The results showed that after 24 h treatment with 10 µM QUE and 200 µM CuSO_4_, the mRNA expression levels of antioxidant enzymes *CAT* and *SOD2* were significantly increased, and the mRNA expression level of antioxidant-related genes *HO-1*, *TRX* was significantly decreased compared with the 200 µM CuSO_4_ treatment group. In addition, the protein expression level of HO-1 was significantly decreased. Although the expression of SOD2 protein did not reach a significant level, it was obviously increased. The contents of MDA and SOD in the cells were significantly decreased, and CAT and GSH were significantly increased. These results suggest that QUE could alleviate CuSO_4_-induced oxidative stress, thereby reducing the cytotoxicity of CuSO_4_ on pGCs.

### 3.5. QUE Alleviated the Mitochondrial Damage Induced by High Copper in pGCs

**Figure 5:**
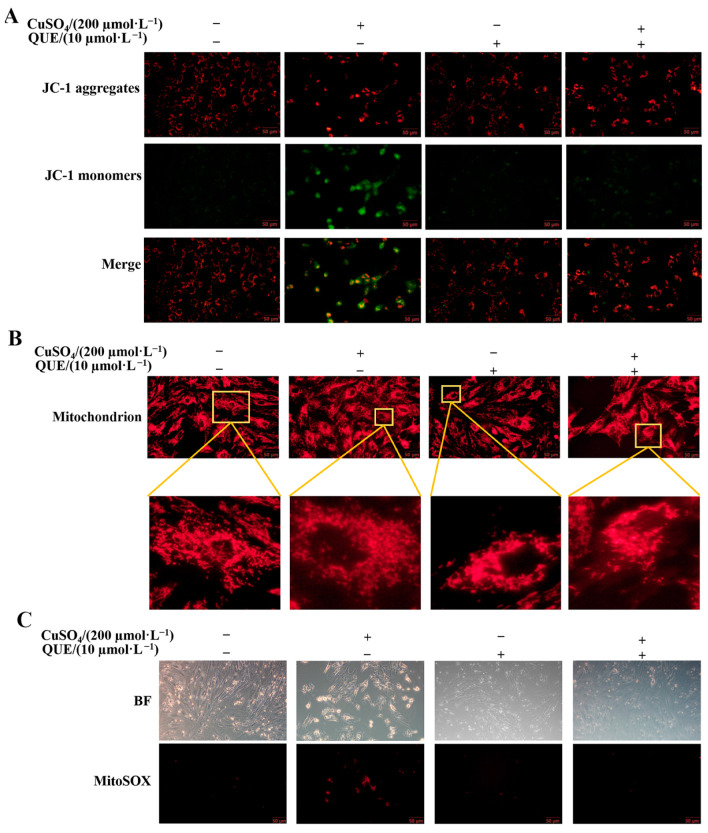
Effect of QUE on high copper-induced mitochondrial oxidative stress and mitochondrial morphology. (**A**) JC-1 fluorescent probe method was used to detect MMP changes in pGCs treated with 10 µM QUE and 200 µM CuSO_4_ co-treated group for 24 h. (**B**) Mitochondrial morphology of pGCs after 24 h combined treatment with 10 µM QUE and 200 µM CuSO_4_. Mitochondria were labeled with MitoTrackerTM. Scale bar: 50 µm. (**C**) mtROS levels were measured by Mito SOX fluorescent probe assay after 24 h of combined treatment with 10 µM QUE and 200 µM CuSO_4_. Scale bar: 100 µm.

After 24 h of combined treatment with 10 µM QUE and 200 µM CuSO_4_, the changes in MMP of pGCs were detected by fluorescence microscopy, as shown in Figure 5A. Compared with the 200 µM CuSO_4_-treated group, the red fluorescence representing JC-1 polymer was enhanced and the green fluorescence representing JC-1 monomer was decreased. These results indicated that QUE markedly reversed the Cu-induced decrease in MMP. In addition, in the 200 µM CuSO_4_-treated group, the mitochondria were fragmented, shortened in length, and even dotted. However, QUE was able to protect the mitochondria to maintain normal reticular formation. The figure (Figure 5B) showed that most of the mitochondria in the 10 µM QUE and 200 µM CuSO_4_ co-treated group were filamentous or tubular in shape, and the number of fragmented mitochondria was significantly reduced (Figure 5B), indicating that the balance of mitochondrial fission and fusion could be disturbed by 200 µM CuSO_4_, which could be alleviated by QUE. In addition, analysis of mtROS levels using fluorescence microscopy showed that QUE was able to reduce mtROS levels (Figure 5C).

## 4. Discussion

Copper is widely used in agriculture and industrial production, and eventually accumulates in large quantities in the ecosystem. Animals are exposed to copper in the environment through feed and water [29]. In addition, CuSO_4_ is added to pig feed for growth promotion [30]. Therefore, pigs are at great risk of copper exposure. Usually, copper is mainly absorbed in the duodenum of pigs. After most of the copper is used by the body, excess copper ions in the organism are accumulated in the liver [31]. However, when copper accumulation exceeds the threshold of the liver, copper is transported through the blood circulation to the ovaries, kidneys, testes, and other tissues and organs, where it is deposited [30]. At present, the target organs of copper toxicity studied mainly focus on the liver and kidney, and there is little research on the reproductive toxicity of copper. In this study, the oxidative toxicity of high copper on pGCs were analyzed. Granulosa cells are the main functional cells in ovary, which play an important role in oocyte maturation, protect the oocyte, and deliver nutrients to the oocyte through gap junctions [32]. Thus, granulosa cell status could affect follicular development and, consequently, the reproductive potential of the dam [33]. Previous studies have shown that copper could accumulate in multiple organs to exert its toxic effects [6,34,35,36]. Babaei et al. showed that long-term or high-dose copper intake could affect ovarian structure [37], but little is known about pGCs. In the present study, a copper toxicity model was established to evaluate the effects of copper exposure on pGCs.

Oxidative stress refers to the excessive production of free radicals such as reactive oxygen species and reactive nitrogen species in the body when the body is subjected to various harmful stimuli. The degree of oxidation exceeds the antioxidant capacity of cells to remove oxides, and the imbalance between the oxidative and antioxidant systems leads to cell damage [38]. Numerous studies have shown that excess copper is able to induce cellular ROS production [8,34,35,39,40]. As a copper ion is a metal ion with strong redox activity, copper could catalyze the Fenton reaction, which is the reaction of divalent iron ion with H_2_O_2_ to produce a large number of hydroxyl radicals. And hydroxyl radicals with strong oxidation can trigger the generation of more oxygen radicals, which is the main mechanism of ROS production induced by copper [14]. The high production of ROS disturbs the body’s redox balance, which is also confirmed in our article, and our results show that copper exerts its cytotoxicity by inducing oxidative stress-mediated mitochondrial damage in pGCs.

In pGCs, copper catalyzes ROS production and increases in a dose-dependent manner. Excessive ROS could attack cellular biomolecules and cause lipid peroxidation [41]. GPX4 is an antioxidant enzyme that inhibits the process of lipid peroxidation. However, in our assay results, it was shown that the mRNA level of the *GPX4* gene was reduced by high copper, which may cause lipid peroxidation. And we detected an increase in MDA, the end product of membrane lipid oxidation, in cells treated with 200 µM CuSO4, indicating that membrane lipids suffered from ROS damage. The generation and accumulation of ROS inhibited the proliferation of pGCs and reduced the activities of the antioxidant enzyme CAT and the antioxidant GSH, which are key indicators of cellular oxidative stress. Among them, SOD could dismutate superoxide anion to H_2_O_2_, CAT could decompress H_2_O_2_ to water and oxygen, and GSH could scavenge ROS and protect other antioxidant enzymes and proteins from oxidation. Interestingly, Cu increased the level of superoxide dismutase (SOD), which is composed of copper-zinc superoxide dismutase (SOD1) and manganese superoxide dismutase (SOD2) in vertebrates [42]. The mRNA and protein levels of SOD2 were detected, and the expression of SOD2 was significantly decreased compared with the control group. The increase in the total SOD content may be due to the fact that copper is the active center of SOD1 [43], the metal prosthetic group of SOD1, and copper is involved in the synthesis of SOD1 [44]. However, the increase in SOD1 cannot completely remove the excess ROS produced by copper catalysis, and could not counteract the imbalance of redox level caused by copper. HO-1 catalyzes the decomposition of heme to exert its antioxidant effect, and is a common downstream product of the Keap1-Nrf2-ARE pathway [45]. When cells undergo oxidative stress, the Keap1-Nrf2-ARE signaling pathway will be activated. Keap1 and Nrf2 will accelerate the dissociation, promote Nrf2 to enter the nucleus and bind to ARE, and activate the transcription of downstream genes [46,47]. In addition, a number of studies related to copper poisoning have shown that excess copper activates the Keap1-Nrf2-ARE signaling pathway, which activates the transcription of *HO-1* and other downstream products in a variety of cells, such as porcine oocytes [37], the kidney tissues of mice [26], and murine hippocampal neuronal cell [17]. In our results, both the mRNA and protein levels of HO-1 were increased after high copper treatment. This is consistent with the results of the literature described above. The *TRX* gene encodes thioredoxin, which plays its antioxidant role by scavenging ROS and reducing disulfide bonds of a variety of proteins. TRX is one of the major redox regulatory molecules in cells, and oxidative stress and other stimuli induce its expression in cells [48]. Therefore, the mRNA level of *TRX* was increased, indicating that the cells received oxidative stress stimulation. To sum up, we could conclude that the Cu-induced ROS generation causes an imbalance in the redox level of pGCs, which eventually leads to oxidative stress in the cells.

Mitochondria is an important organelle for generating energy, maintaining calcium homeostasis, producing ROS, and regulating apoptosis [49,50,51]. Alterations in mitochondrial morphology and function are associated with the cytotoxicity of copper. Damaged mitochondria not only fail to produce energy efficiently, but also excessively accumulate ROS [52], leading to Ca^2+^ imbalance, which may exacerbate cell damage caused by high copper. We examined the mtROS content in our experiments, and as expected, the mtROS level increased with the increase in high CuSO_4_ concentration (≥200 µM). In the following experiments, it was also found that high copper was able to reduce the MMP of pGCs, indicating that mitochondrial membrane permeability is altered and mitochondrial proteins are released to the cytoplasm. In addition, MMP is also a hallmark event in the early stage of apoptosis, indicating that the cells are evolving toward the outcome of apoptosis. At the same time, transmission electron microscopy results also showed that the membrane structure of mitochondria was damaged, the morphology became swollen, and there were even empty areas in some mitochondria. Furthermore, normal mitochondria are distributed in a network structure in the cell; however, because the morphology of mitochondria is changed from a short rod to a dot shape by high copper, we can find that the network structure of mitochondria is also disrupted. What is more important is that these changes in mitochondrial morphology are often accompanied by mitochondrial dysfunction. Based on the above results, we concluded that copper catalyzes ROS production, damages mitochondria, and leads to mitochondrial dysfunction, which ultimately exerts a toxic effect on pGCs. Thus, the model of copper toxicity in pGCs is successfully established.

Copper catalyzes oxidative stress in pGCs, resulting in cell damage. Therefore, finding an ingredient that could effectively inhibit the excessive production or accumulation of ROS catalyzed by copper is an important method to alleviate copper toxicity.

QUE is widely found in vegetables, fruits, and common Chinese herbs. It is a flavonoid with a wide range of biological activities such as antioxidation, anti-apoptosis, and anti-inflammation [53,54,55,56]. It has been reported that QUE protects granulosa cells from damage caused by oxidative stress in pigs [25], goats [57], buffaloes [28], and chickens [58]. Moreover, QUE alleviates the toxic effects of heavy metals such as copper [59], cadmium [60], cisplatin [61], iron [62], and lead [63]. And a physiological dose of QUE could remove ROS and effectively reverse the decrease in MMP [64,65,66], thus exerting its antioxidant effect to protect cells. This was confirmed in our experiment, where after the addition of 10 µM QUE, we found that the cell activity of pGCs was increased, the antioxidant capacity was also restored to the normal level, and the level of ROS and the degree of membrane lipid peroxidation in cells were decreased compared with the 200 µM CuSO_4_-treated group. That indicated that QUE could alleviate CuSO_4_-induced oxidative stress by scavenging ROS. Moreover, the MMP was increased, the mitochondrial reticular structure was restored, and the level of mtROS was decreased after the addition of QUE, suggesting that QUE could protect mitochondria from high copper-induced oxidative stress-mediated mitochondrial damage. So far, we have only verified that oxidative stress is one of the toxic mechanisms of high copper on pGCs, and QUE protects pGCs by alleviating oxidative stress. However, the mode of oxidative stress-induced cell death and the protective effect of QUE on the cell death mechanism have not been thoroughly investigated. To address this deficiency, we will further investigate the mode of death mediated by oxidative stress induced by high copper and the protective function of QUE based on this study in order to further understand the molecular mechanism of reproductive toxicity induced by high copper.

## 5. Conclusions

In summary, our study showed that high copper has oxidative toxicity to pGCs and QUE can clear ROS induced by high copper, protect mitochondria from oxidative stress, improve the function of pGCs. And it is expected to reduce porcine follicular atresia and protect the reproductive ability of sows.

## Figures and Tables

**Table 1:** Reaction procedure for qRT-PCR.

Step	Temperature	Time	Cycle Number
Predenaturation	95 °C	1 min	1
Denaturation	95 °C	20 s	40
Annealing	60 °C	20 s	
Extension	72 °C	30 s	

**Table 2:** Primers used for qRT-PCR.

GenBank Accession Number	Gene Name	Primer Sequence (5′→3′)	Anneal T (°C)	Product Size (bp)
NM_001206359	*GAPDH*	F: TCGGAGTGAACGGATTTGGC R: TGCCGTGGGTGGAATCATAC	60	147
NM_214407	*GPX4*	F: GACGACTGGCGATGTGCT R: GCTCCTGCCTCCCAAACT	60	232
NM_001190422	*SOD1*	F: ATTCTGTGATCGCCCTCT R: AGCATTTCCCGTCTTTGT	60	119
NM_214127	*SOD2*	F: TCTGGACAAATCTGAGCCCTAA R: TGGACGCCGACGGATACA	60	127
XM_021081498	*CAT*	F: CGAAGGCGAAGGTGTTTG R: CAAACCCACGAGGGTCAC	60	114
NM_001004027	*HO-1*	F: GCTGAGAATGCCGAGTTCAT R: GGAAGTAGAGGGGCGTGTAG	60	157
XM_021083263	*TRX*	F: TTCCAATGTCGTGTTCCTTG R: ACCCACCTTCTGTCCCTTTT	55	115

## Data Availability

The original data in the article can be obtained directly from the corresponding author.

## Supplementary Materials

The following supporting information can be downloaded at: https://www.mdpi.com/article/10.3390/ani13172745/s1, Western Blot (WB) results of Figure 2C,E and Figure 4F,H.
