# Supplementary material for: Quercetin Alleviates Toxicity Induced by High Levels of Copper in Porcine Follicular Granulosa Cells by Scavenging Reactive Oxygen Species and Improving Mitochondrial Function
Source: Animals (Basel). 2023 Aug 29;13(17):2745. doi: 10.3390/ani13172745 (PMC10486440; doi:10.3390/ani13172745)
Supplement: Supplementary file 1 [file animals-13-02745-s001.zip › animals-2530403-supplementary.pdf]

Figure 2C

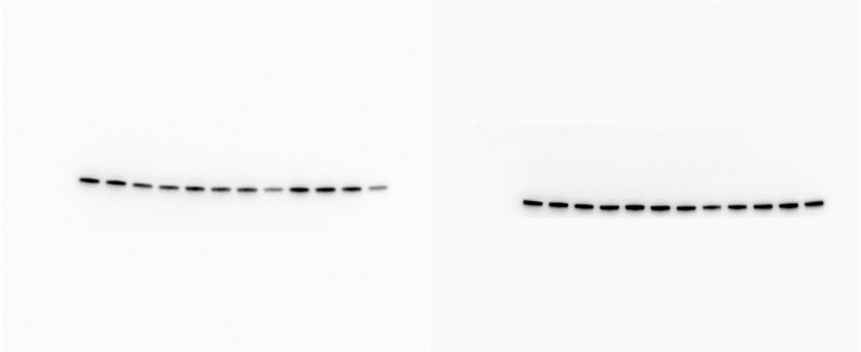

SOD2 (25 KDa) TUBULIN(55 KDa)

Figure 2E

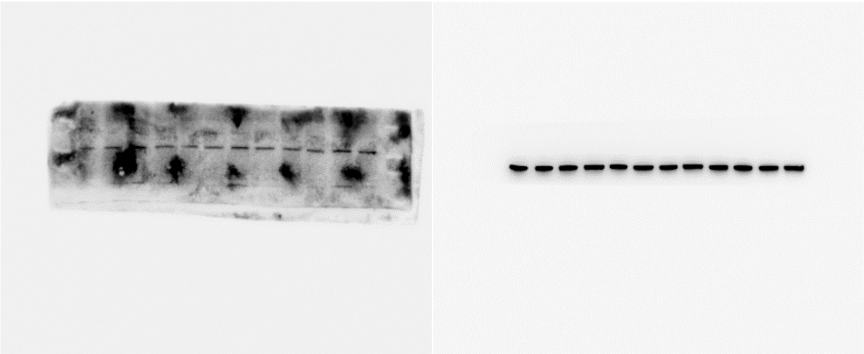

HO-1 (32 KDa) TUBULIN(55 KDa)

In the above two groups of pictures, lanes 1, 5, and 9 are the control group; lanes 2, 6, and 10 are the 100μM copper sulfate treatment group; lanes 3, 7, and 11 are the 200μM copper sulfate treatment group; lanes 4, 8, and 12 are the 400μM copper sulfate treatment group.

Figure 4F

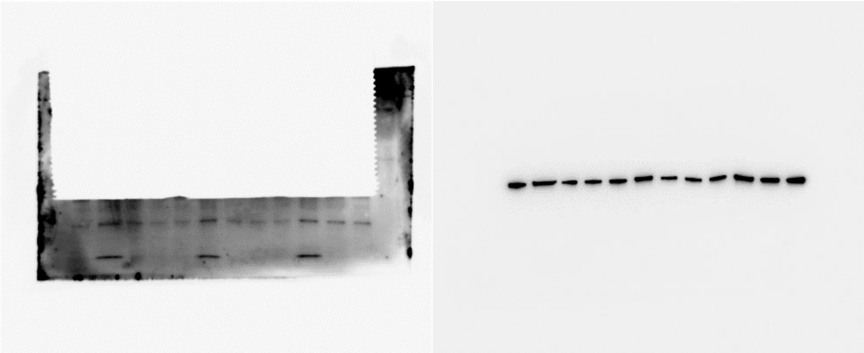

HO-1 (32 KDa) TUBULIN (55 KDa)

In the above group of pictures, lanes 1, 5, and 9 are the control group; lanes 2, 6, and 10 are the 200μM copper sulfate treatment group; lanes 3, 7, and 11 are the 10μM quercetin treatment group; lanes 4, 8, and 12 are the 200μM copper sulfate combined with 10μM quercetin treatment group.

Figure 4H

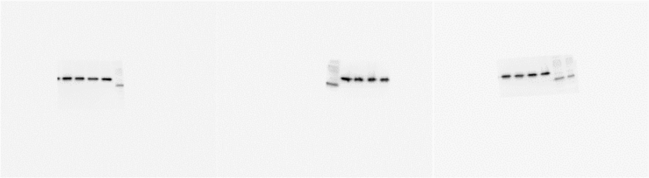

SOD2 (25 KDa)

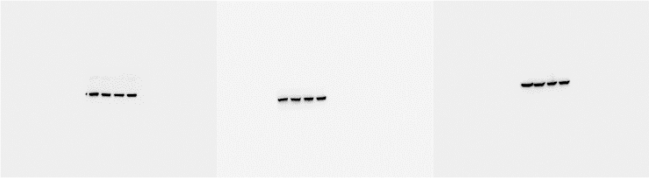

TUBULIN (55 KDa)

In the above group of pictures, lane 1 is the control group; lane 2 is the 200μM copper sulfate treatment group; lane 3 is the 10μM quercetin treatment group; lane 4 is the 200μM copper sulfate combined with 10μM quercetin treatment group.
